# Artificial intelligence for morphology-based function prediction in neovascular age-related macular degeneration

**DOI:** 10.1038/s41598-019-47565-y

**Published:** 2019-07-31

**Authors:** Leon von der Emde, Maximilian Pfau, Chantal Dysli, Sarah Thiele, Philipp T. Möller, Moritz Lindner, Matthias Schmid, Monika Fleckenstein, Frank G. Holz, Steffen Schmitz-Valckenberg

**Affiliations:** 1 Department of Ophthalmology, University of Bonn, Ernst-Abbe-Str. 2, Bonn, Germany; 2 GRADE Reading Center, Ernst-Abbe-Str. 2, Bonn, Germany; 3 Department of Ophthalmology and Department of Clinical Research, Inselspital, Bern University Hospital and University of Bern, Bern, Switzerland; 4 The Nuffield Laboratory of Ophthalmology, Sleep and Circadian Neuroscience Institute, Nuffield Department of Clinical Neurosciences, University of Oxford, Oxford, United Kingdom; 5 Institute for Medical Biometry, Informatics and Epidemiology, University of Bonn, Bonn, Germany

**Keywords:** Predictive markers, Predictive markers

## Abstract

Spatially-resolved mapping of rod- and cone-function may facilitate monitoring of macular diseases and serve as a functional outcome parameter. However, mesopic and dark-adapted two-color fundus-controlled perimetry (FCP, also called “microperimetry”) constitute laborious examinations. We have devised a machine-learning-based approach to predict mesopic and dark-adapted (DA) retinal sensitivity in eyes with neovascular age-related macular degeneration (nAMD). Extensive psychophysical testing and volumetric multimodal retinal imaging data were acquired including mesopic, DA red and DA cyan FCP, spectral-domain optical coherence tomography and confocal scanning laser ophthalmoscopy infrared reflectance and fundus autofluorescence imaging. With patient-wise leave-one-out cross-validation, we have been able to achieve prediction accuracies of (mean absolute error, MAE [95% CI]) 3.94 dB [3.38, 4.5] for mesopic, 4.93 dB [4.59, 5.27] for DA cyan and 4.02 dB [3.63, 4.42] for DA red testing. Partial addition of patient-specific sensitivity data decreased the cross-validated MAE to 2.8 dB [2.51, 3.09], 3.71 dB [3.46, 3.96], and 2.85 dB [2.62, 3.08]. The most important predictive feature was outer nuclear layer thickness. This artificial intelligence-based analysis strategy, termed “inferred sensitivity”, herein, enables to estimate differential effects of retinal structural abnormalities on cone- and rod-function in nAMD, and may be used as quasi-functional surrogate endpoint in future clinical trials.

## Introduction

Age-related macular degeneration (AMD) is the most common cause for severe visual loss in industrialized countries^1^. The introduction of anti-vascular endothelial growth factor (anti-VEGF) therapy has markedly improved visual outcomes in patients with choroidal neovascularization (CNV) secondary to AMD^2^. However, clinical trials investigating combined therapeutic approaches (e.g. anti-VEGF in combination with anti- Pigment epithelium-derived factor (PEDF) beyond anti-VEGF monotherapy have yet failed to demonstrate superiority (e.g. CAPELLA, [clinicaltrials.gov identifier: NCT02418754], OPH1002 [NCT01944839], OPH1003 [NCT01940900])^3^. While the negative trial results may be explained by the lack of biological effectiveness, they may also be a result of limitations of the utilized structural surrogate and functional endpoints. In a worst-case scenario, this may lead to disregarding a candidate drug that in fact actually was efficient.

Best-corrected visual acuity (BCVA) is the most commonly used functional endpoint in ophthalmological trials. However, it has limited accuracy with regard to subtle therapeutic effects, as it only measures photopic function at central retinal fixation and exhibits considerable retest-variability^4,5^. In this regard, fundus-controlled perimetry (FCP, ‘microperimetry’) offers information over and beyond BCVA. FCP is a established psychophysical assessment allowing for spatially resolved probing of retinal sensitivity even in patients with instable fixation due to eye tracking^6–10^. Recently, the refined probing of rod function by dark-adapted (DA) two-color FCP has become possible with the introduction of a novel device (S-MAIA, Centervue, Padua, Italy)^11–14^. However, the test requires dedicated equipment, is rather time consuming and the number of test-points and consequently the spatial-resolution is limited due to fatigue of the patient. Spectral-domain optical coherence tomography imaging (SD-OCT), which allows for axially resolved imaging of the retina, infrared reflection (IR) imaging and fundus autofluorescence (FAF) imaging, which enables mapping of retinal fluorophores, are now widely available^15^. Hereby, the *en face* resolution of these modalities (11.4 or 5.7 µm/pixel for the Spectralis OCT 2 device, Heidelberg Engineering, Germany) is by more than one log unit higher as compared to FCP testing (128 µm [Goldmann III] stimulus for the S-MAIA device). However, the currently used imaging biomarkers have limited informative value. For example in neovascular AMD, a decrease in central (full) retinal thickness could represent both, positive (e.g. reduction of macular edema) or negative (e.g. outer retinal atrophy) treatment effects. It has recently been demonstrated, that artificial intelligence (AI) algorithms, including machine learning techniques such as random forest regression, may be applied in neovascular AMD to predict future BCVA based on previous BCVA and structural SD-OCT data^16^. Yet, similar to BCVA, “inferred BCVA” would be expected to be rather insensitive to localized - particularly extrafoveal - alterations in the retinal structure.

The aim of this study was to predict retinal senstivity based on retinal microstructure in neovascular AMD using machine-learning algorithms. The analysis was based on multimodal, volumetric state-of-the-art retinal imaging and differential mesopic, dark-adapted cyan and dark-adapted red FCP testing. To potentially improve the accuracy of the applied models, we also estimated the additional predictive value provided by “patient-reliability indices” that may account for patient-specific behavioral factors. Finally, we designed this study with the aim to explore the utility of “inferred sensitivity” mapping as a quasi-functional surrogate endpoint for future clinical trials. Hereby, we introduce the term “inferred sensitivity” to describe the spatially-resolved prediction of retinal sensitivity based on clinically feasible multimodal retinal imaging and with subsequent application of AI algorithms.

## Results

### Cohort characteristics

**Figure 1:**
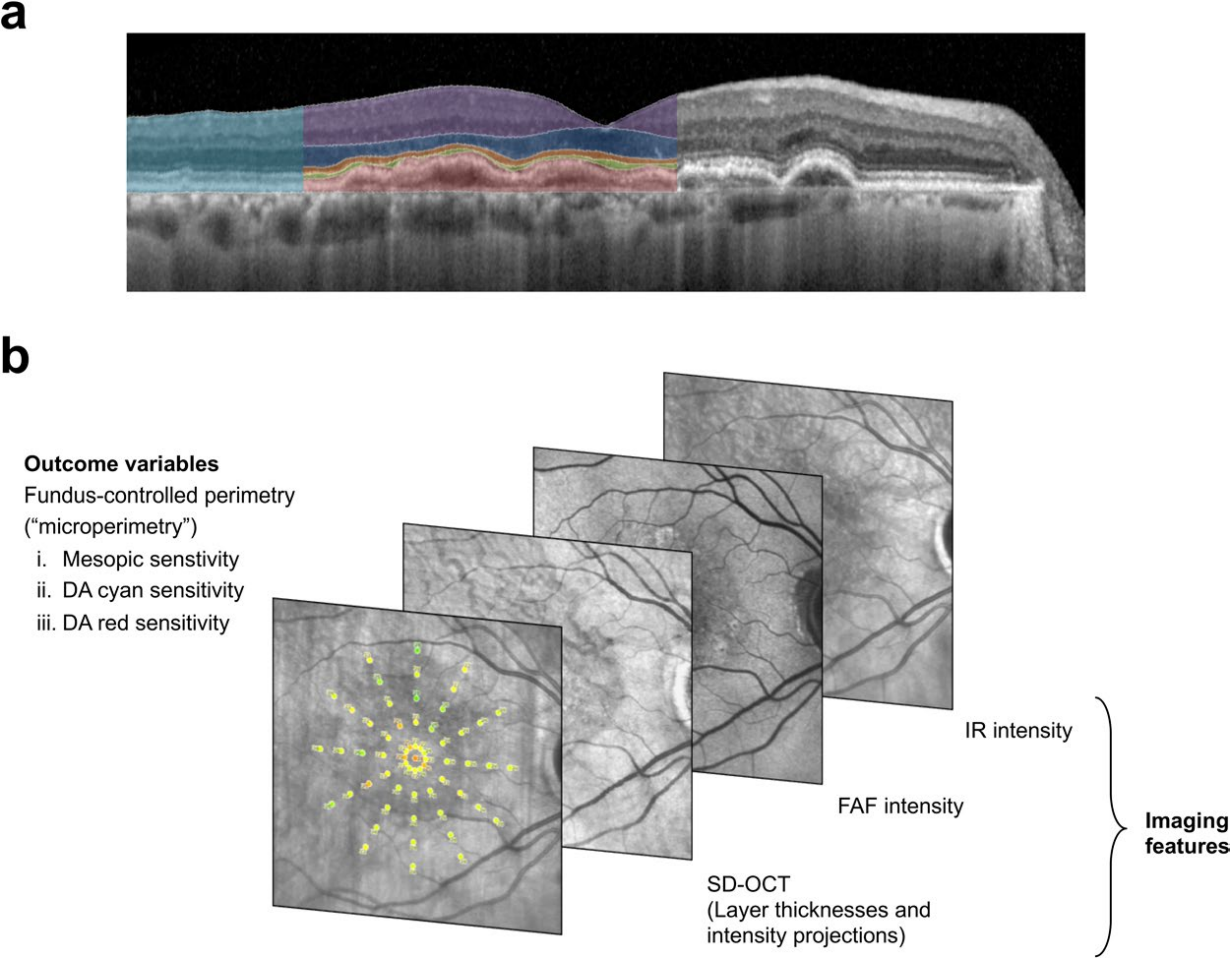
Image registration and grading. As shown in panel A, spectral-domain optical coherence tomography was semi-automatically annotated. The herein used definition for the full-retina (turquoise overlay) ranged from the internal limiting membrane (ILM) to Bruch’s membrane (BM), the inner retina (purple overlay) from the ILM to the outer plexiform layer (OPL) outer nuclear layer (ONL) boundary, the ONL (blue layer) from the OPL/ONL boundary to the external limiting membrane (ELM), the inner photoreceptor segments (IS, red overlay) from the ELM to band 2 (ellipsoid zone [EZ]), the outer photoreceptor segments (OS, green overlay) from EZ to band 3 (interdigitation zone) or upper boundary of the retinal-pigment-epithelium-drusen-complex (RPEDC, red overlay). RPE, drusen, reticular drusen and subretinal hyperreflective material were defined as RPEDC. Subretinal fluid was included in the here used definition of the OS layer. As shown in panel B, fundus-controlled perimetry data was registered to the multimodal imaging data based on vessel bifurcations to extract imaging features corresponding precisely to the test-point location and area (0.43°). For each test-point, 26 imaging features (fundus autofluorescence [FAF] intensity, infrared reflection [IR] intensity as well as thickness, minimum- mean- and maximum-intensity projections for the SD-OCT layers [full retina, inner retina, ONL, IS, OS, RPEDC]) were extracted.

Fifty eyes of 50 patients with CNV secondary to AMD (age [mean ± SD] 76.1 ± 7.6 years [range: 54.6–90.2 years]) and 40 eyes of 40 controls (55.8 ± 17.4 years [21.8–82.1 years]) were included in this study (Table 1). The median BCVA was logMAR 0.38 ± 0.34 [Snellen equivalent approximately 20/50] for patients and 0.03 ± 0.07 [Snellen equivalent approximately 20/20] for controls. For all following analyses, the normal data was exclusively used to standardize patient data in consideration of the spatial differences in retinal sensitivity as well as layer thicknesses and reflectivities (cf. Methods and Fig. 1). Accordingly, only patient data were used to derive the estimates for the prediction accuracies to obtain as much as possible conservative estimates. A single observation (i.e. single test-point within one patient) for all three types of FCP testing had to be excluded due to missing SD-OCT data. This left a total of 3049 observations for predictive modeling for each type of testing (i.e. 50 patients with 61 point-wise observations for mesopic, DA cyan and DA red testing).

**Table 1:** Cohort characteristics.

Characteristic	Overall cohort (N = 50)	Retest subgroup (N = 28)
Age (mean ± SD)	76.1 ± 7.6 years	76.5 ± 7.3 years
Sex	30 female	18 female
	20 male	10 male
BCVA (mean ± SD)	0.38 ± 0.34 logMAR	0.38 ± 0.33 logMAR
CNV subtype	32 active CNV	18 active CNV
	11 silent CNV	6 silent CNV
	7 quiescent CNV	4 quiescent CNV
**Retest reliability (MAE [95% CI])**		
Mesopic testing	n/a	2.00 dB [1.81, 2.19]
Dark-adapted cyan testing		1.89 dB [1.56, 2.22]
Dark-adapted red testing		2.20 dB [1.95, 2.46]

The cohort characteristics are outlined. The randomly sampled subgroup of patients that underwent duplicate testing is highly representative of the overall cohort. The mean absolute error (MAE) between the first and second test for the retest subgroup were provided as a benchmark for the prediction results shown in Table 2.

### Prediction model for retinal sensitivity in an unknown patient (scenario 1)

**Figure 2:**
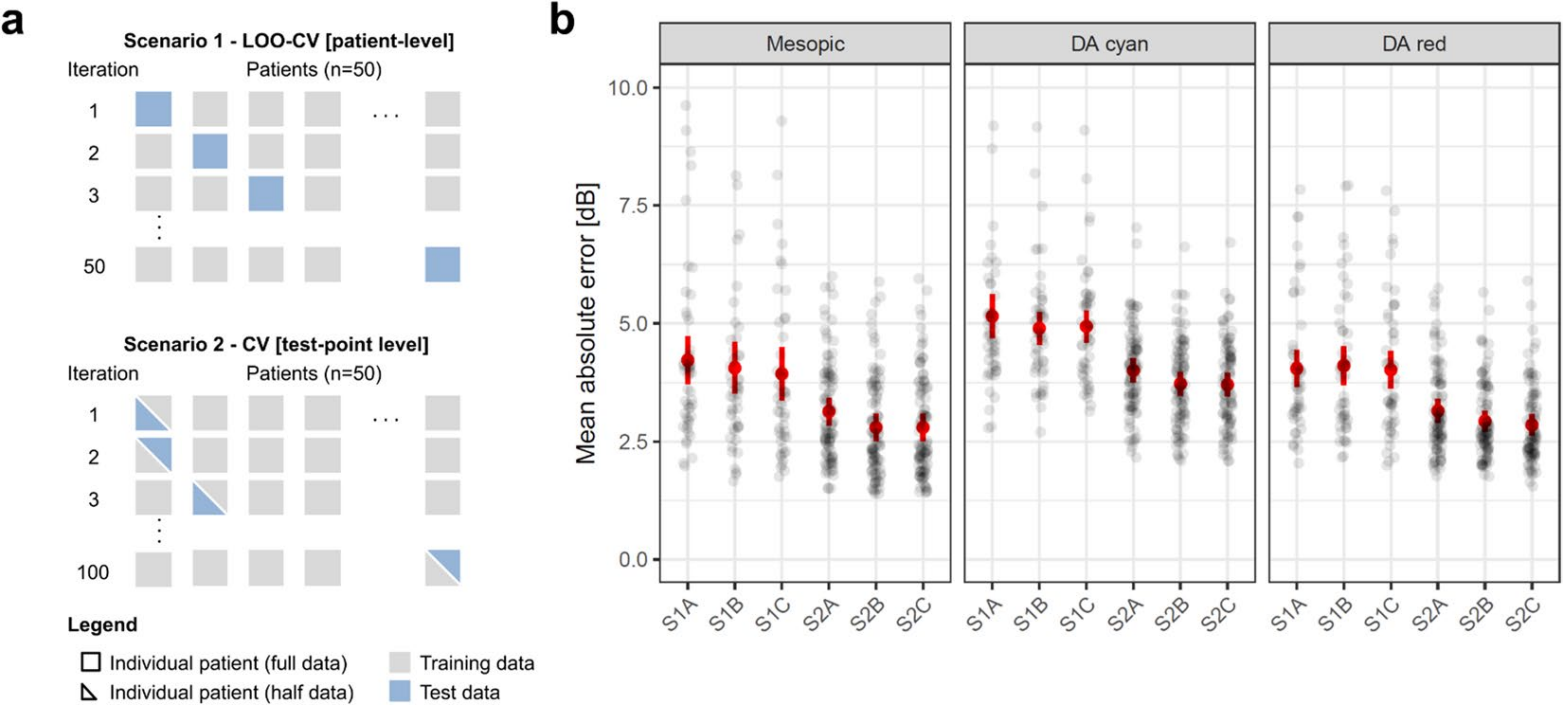
Cross-validation and prediction accuracies. Panel A shows the “outer” resampling techniques used in this study to estimate the efficacy of the models. First, we used patient-wise leave-one-out cross-validation (LOO-CV) to estimate the prediction accuracy for an “unknown patient” (scenario 1 [S1]). Second (scenario 2 [S2]), we evaluated the prediction accuracy for a patient with some available perimetry data (i.e. data of a brief exam that would be feasible in a multicentre trial [30 test-points, duration of approximately 5 minutes]). Both scenarios were probed with three different sets of predictor variables: (S1A or S2A) imaging data only, (S1B or S2B) imaging data and “patient reliability indices” and (S1C or S2C) imaging data, “patient reliability indices” and fixation stability. Panel B shows the mean absolute errors (MAE) between predictions and observations as a measure of prediction accuracy per patient in the background for the three types of testing (mesopic, dark-adapted [DA] cyan and dark-adapted red). The red dots indicate the overall MAE (error bars indicate the 95% confidence intervals for the estimate of the MAE).

The prediction accuracies of the machine learning models were determined in two clinically meaningful scenarios. Firstly, scenario 1 (patient-wise leave-one-out cross-validation [LOO-CV], Fig. 2) represents the prediction accuracy for a completely unknown patient with only imaging data available. For mesopic sensitivity, the prediction accuracy for scenario 1 based on imaging data only (S1A) reached a mean absolute error (MAE [95% CI]) of 4.22 dB [3.72, 4.72], which is markedly better as compared to the MAE of the corresponding null model (Table 2). A likelihood ratio test revealed, that the prediction accuracies varied significantly in dependence of the feature set (P < 0.001). With additional inclusion of “patient reliability indices” as predictors (S1B), to control for potential confounding factors such as the patient-specific false-positive response rate, the MAE could be further reduced to 4.06 dB [3.52, 4.6] (P < 0.001). Of note, fixation stability was not considered as “patient reliability indices” in this study, since it could be partially informative of function. Additional inclusion fixation stability (S1C) allowed for a further slight reduction of the MAE to 3.94 dB [3.38, 4.5] as shown in Fig. 2 (P < 0.001). Similar prediction accuracies were reached for S1A for dark-adapted cyan sensitivity (5.15 dB [4.68, 5.62]) and dark-adapted red testing (4.05 dB [3.66, 4.43]). Again, the prediction accuracy varied in dependence of the feature set for both types of testing (likelihood ratio test, P < 0.001). Hereby, inclusion of “patient reliability indices” likewise improved the prediction accuracies markedly for dark-adapted cyan testing to 4.89 [4.55, 5.24] (P < 0.001). For dark-adapted red testing, the prediction accuracy did not improve through inclusion of “patient reliability indices” as predictors. Inclusion of fixation stability did not further improve the prediction accuracies significantly for both types of testing (Fig. 2, Table 2).

**Table 2:** Prediction accuracies.

	Scenario 1: Leave One Out Cross Validation (LOO-CV) on patient-level				Scenario 2: Cross Validation (CV) on test-point level			
		Random forest regression				Random forest regression		
Type of testing	Null model	Imaging data	Imaging data and reliability indices	Imaging data and reliability indices and fixation stability	Null model	Imaging data	Imaging data and reliability indices	Imaging data and reliability indices and fixation stability
Mesopic (MAE in dB)	5.29 [4.59, 6.00]	4.22 [3.72, 4.72]	4.06 [3.52, 4.6]	3.94 [3.38, 4.5]	3.74 [3.16, 4.31]	3.14 [2.85, 3.43]	2.8 [2.51, 3.09]	2.8 [2.51, 3.09]
Dark-adapted cyan (MAE in dB)	6.69 [6.25, 7.13]	5.15 [4.68, 5.62]	4.89 [4.55, 5.24]	4.93 [4.59, 5.27]	4.94 [4.6, 5.28]]	4.01 [3.76, 4.26]	3.73 [3.48, 3.98]	3.71 [3.46, 3.96]
Dark-adapted red (MAE in dB)	5.39 [4.76, 6.02]	4.05 [3.66, 4.43]	4.11 [3.7, 4.51]	4.02 [3.63, 4.42]	3.82 [3.41, 4.23]	3.15 [2.9, 3.41]	2.93 [2.71, 3.16]	2.85 [2.62, 3.08]

The mean absolute error (MAE) estimates and 95% confidence interval for the MAE estimates are provided as measure of prediction error.

### Prediction model for retinal sensitivity in a patient with prior perimetry data (scenario 2)

Since potentially influential factors (e.g. lens opacity) may not be directly deducible from retinal imaging data, we assessed whether data from a brief FCP exam, which would be feasible in the context of a multicenter trial, (i.e. 30 test-points, duration of 5 minutes), could further enhance the prediction accuracy. For all three types of testing, the prediction accuracy was markedly improved for scenario 2 as compared to scenario 1 (P < 0.001). For all three types of testing, the prediction accuracy varied in dependence of the feature set (likelihood ratio test, P < 0.001). For mesopic sensitivity, the prediction accuracy for scenario 2 reached a MAE of 3.14 dB [2.85, 3.43]. Inclusion of “patient reliability indices” did further reduce the MAE (2.8 dB [2.51, 3.09]; P < 0.001), significantly while additional inclusion of fixation stability as predictor did not result in a further improvement of the prediction accuracy. Similarly, good prediction accuracies were reached for scenario 2A for dark-adapted cyan sensitivity (4.01 dB [3.76, 4.26]) and dark-adapted red testing (3.15 dB [2.9, 3.41]). Inclusion of “patient reliability indices” markedly improved the prediction accuracy for dark-adapted cyan sensitivity (3.73 [3.48, 3.98]; P < 0.001) and dark-adapted red testing (2.93 dB [2.71, 3.16]; P < 0.001). While inclusion of fixation stability as additional predictor did not further improve the prediction accuracy for dark-adapted cyan testing, the prediction accuracy for dark-adapted red-testing was further improved (2.85 dB [2.62, 3.08]; P < 0.001).

### Feature importance and cone versus rod dysfunction

**Figure 3:**
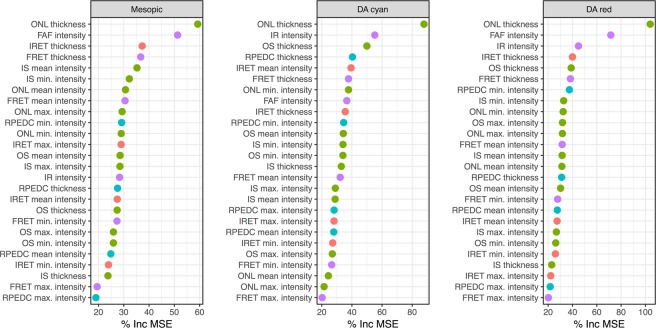
Feature importance. The panels display for all three types of testing the permutation accuracy as measure of feature importance (percentage increase in *mean squared error* [% Inc MSE]). Hereby, the outer nuclear layer (ONL) thickness was the most predictive feature for all three types of testing. Generally, thickness measurements exhibited higher feature importance as compare to the layer intensities. Abbreviations: infrared reflection (IR); retinal-pigment-epithelium-drusen-complex (RPEDC); photoreceptor outer segments (OS); inner segments (IS); inner retina (IRET); full retina (FRET); fundus-autofluorescence (FAF). The color indicates the putative structural correlates of the imaging features (red: inner retina; green: photoreceptors; blue: RPEDC; purple – summation images). Notably, information from all layers contributed towards the predictions.

Based on the permutation accuracy importance, ONL thickness (59.3% Inc MSE) followed by FAF intensity (51.3% Inc MSE) and inner retinal thickness (37.3% Inc MSE) constituted the most important features in predicting mesopic sensitivity (Fig. 3). For prediction of dark-adapted cyan sensitivity, ONL thickness (87.8% Inc MSE) followed by IR intensity (55.2% Inc MSE) and OS thickness (49.9% Inc MSE) constituted the most important features. The feature importance order for dark-adapted red testing was similar to the feature importance for mesopic testing with ONL thickness (103.91% Inc MSE) followed by FAF intensity (71.5% Inc MSE) and IR intensity (44.9% Inc MSE). Moreover, graphical analysis (Fig. 3) underscores that the ONL thickness truly stands out in terms of feature importance across all three types of testing and similarly FAF intensity is separated in terms of importance from the other predictors for mesopic and dark-adapted red testing. Generally, thickness measurements exhibited a higher feature importance as compared to the layer intensities (Fig. 3).

### Structure-function correlation

**Figure 4:**
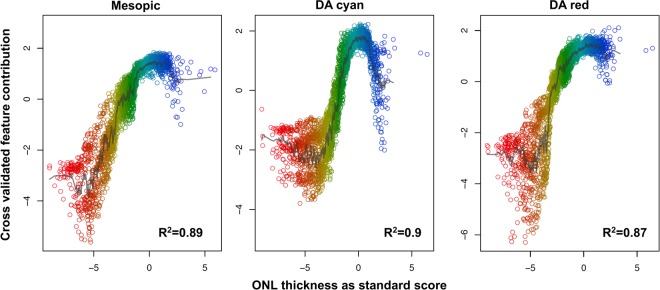
Differential effects of outer nuclear layer (ONL) thickness on cone versus rod function. The panels show the cross-validated feature contribution (y-axis) for mesopic, dark-adapted (DA) cyan and DA red testing in dependence of outer nuclear layer thickness (ONL thickness, x-axis), the imaging feature with the highest predictor importance in all models. For visual purposes, the ONL thickness was further accentuated by the color gradient. Hereby, the ONL thickness is displayed as standard score in consideration of the spatial variability of the ONL thickness (i.e. the number of normative standard deviations by which a given observations deviates from the normative mean estimate). The goodness-of-visualization (R^2^) indicates that the high dimensional model structure can be well reconstructed from the shown low dimensional visualizations. Please note, for all three types of testing, ONL thinning was associated with reduced sensitivity, while normative ONL thickness was associated with normative function. Notably, only for DA cyan testing, the relationship between sensitivity and ONL thickness is inverted-U shaped. This suggests that DA cyan sensitivity is more severely susceptible to ONL thickening (i.e. macular edema) as compared to mesopic or DA red sensitivity.

The projections of feature contributions revealed distinctly different relationships between the retinal sensitivity and the ONL thickness for mesopic testing versus dark-adapted cyan testing (Fig. 4). For both types of testing, reduced ONL thickness indicating outer retinal atrophy was associated with decreased sensitivity, while normative ONL thickness was associated with normal function (Fig. 4). For mesopic and dark-adapted testing, ONL thickening exhibited no distinct effect on sensitivity. In contrast, an inverted-U relationship was observed for dark-adapted cyan sensitivity implying that ONL thickening is associated with a decreased dark-adapted cyan sensitivity (Fig. 4).

### Prediction accuracy and clinical utility

**Figure 5:**
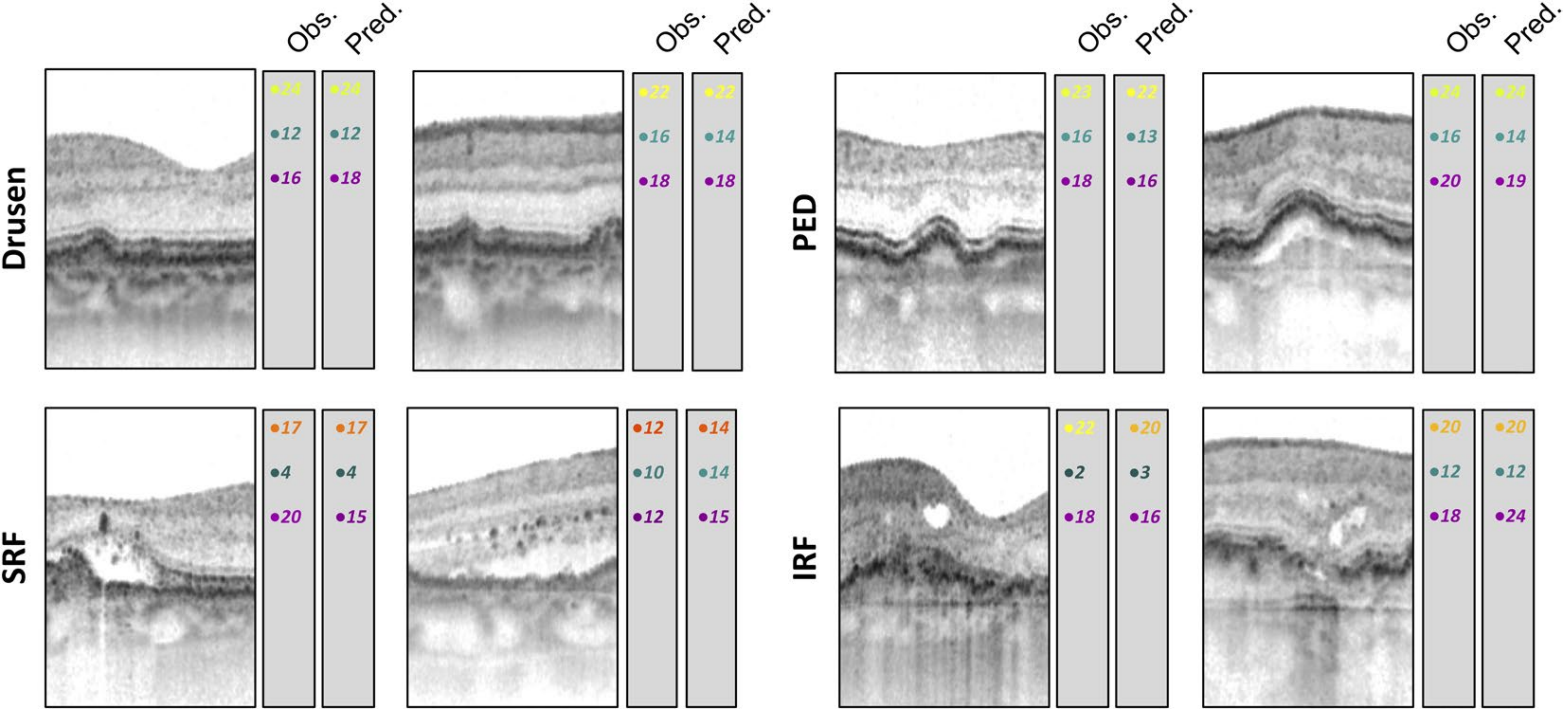
Prediction accuracy and differential effects of disease-associated lesions on cone versus rod function. The panels show exemplary spectral-domain optical coherence tomography for various lesion subgroups (retina with drusen, pigment epithelium detachment [PED], subretinal fluid [SRF], intraretinal fluid [IRF]). For each type of lesion a para-foveal and peripheral B-scan is provided as example. The measured sensitivities as well as the predicted sensitivities for mesopic, dark-adapted (DA) cyan and DA red testing are provided. Please note that DA cyan sensitivity was always lower (observations and predictions) for the para-foveal regions compared to the peripheral regions in agreement with the rod photoreceptor distribution. However, also in the peripheral regions, the (observed and predicted) sensitivities for dark-adapted cyan testing tend to be lower than the sensitivities for dark-adapted red testing indicating selective rod photoreceptor vulnerability.

**Figure 6:**
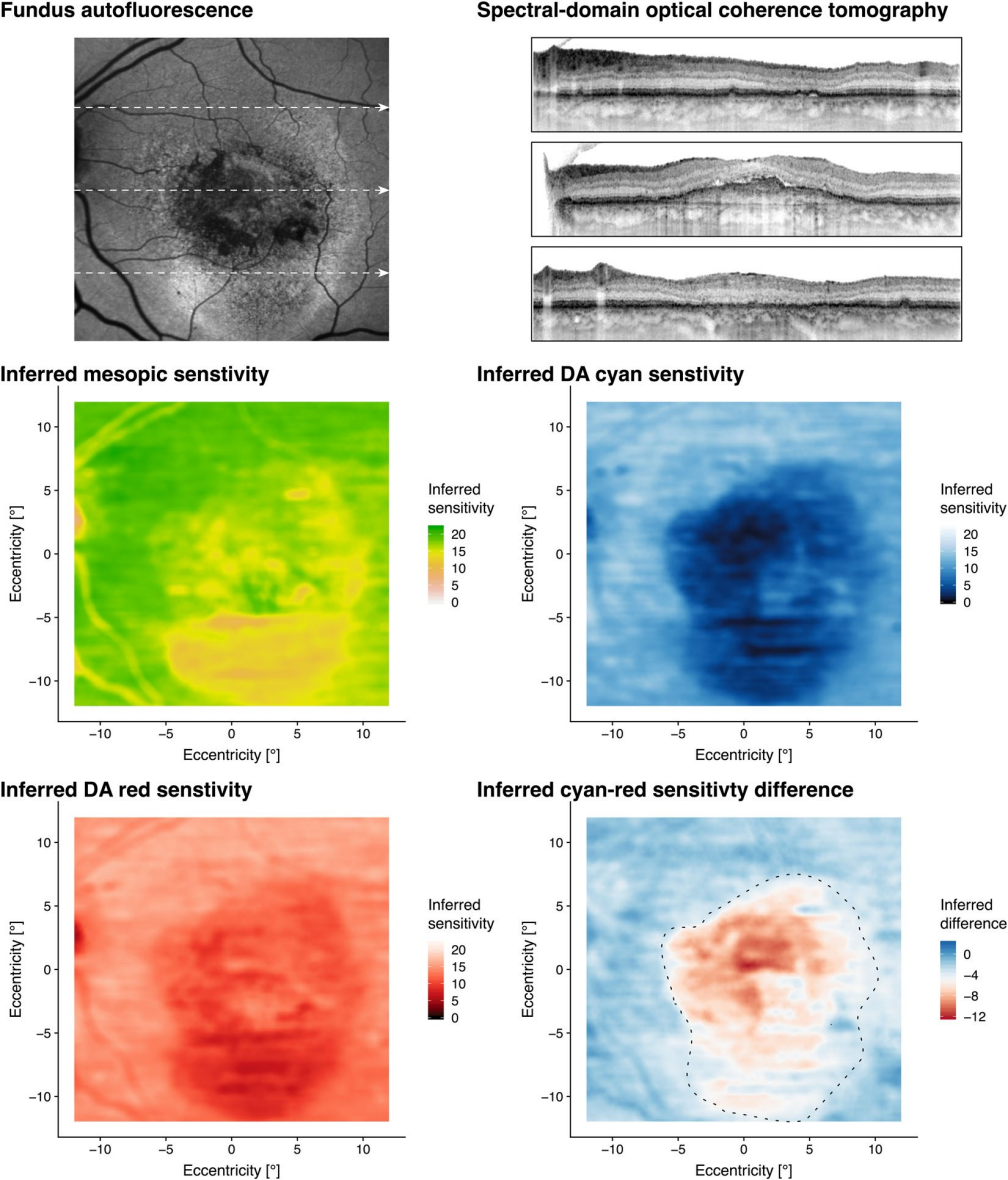
Inferred sensitivity mapping. Based on the fundus autofluorescence (FAF), infrared reflection (IR, not shown) and spectral-domain optical coherence tomography (SD-OCT), mesopic as well as dark-adapted (DA) cyan and DA red sensitivity may be reliably predicted and topographically mapped. The arrows in the FAF image indicate the position of the SD-OCT B-scans. Multiple lines of evidence (besides of the quantitative analysis in Fig. 2) further support the accuracy of the predictions. For all three types of testing, angioscotoma are adequately predicted. Further, the central rod-free zone is also correctly predicted as indicated by the marked cyan-red sensitivity difference at the fovea (eccentricity of 0°, middle B-scan). Regions exhibiting increased FAF and absence of photoreceptor outer and inner-segments (upper and lower SD-OCT scan) show reduced function for all three types of testing. Yet, globally the degree of DA cyan dysfunction appears to exceed the degree of DA red dysfunction. Please note, that the inferred cyan-red sensitivity difference in the region of severe cone dysfunction (delimited by the dashed line) is an underestimation of the true cyan-red sensitivity difference due to the floor effects of the perimetry device used in this study that are inevitable reflected by the models.

Random forest (RF) regression allowed for accurate prediction of sensitivity for a wide variety of retinal structural alterations through the examination of their specific thickness- and reflectivity deviations (Fig. 5). This was confirmed for both, para-foveal as well as peripheral region enabling a comprehensive prediction throughout the retina. Moreover, the algorithm could be used to predict the function for the whole imaged retina (Fig. 6). The patient in Fig. 6 exhibits centrally rather intact retinal sensitivity despite markedly increased central (full) retinal thickness while exhibiting parafoveally scotomata associated with slight retinal thinning. This patient clearly showcases the limitations of central full retinal thickness as surrogate endpoint since full retinal thinning could represent both, loss of function (outer retinal atrophy) and gain of function (reduction in retinal edema).

## Discussion

Demonstrating therapeutic benefits of emerging combined treatment approaches tackling diferent pathways simultaneously constitutes a challenge, especially given that visual outcomes in patients with neovascular AMD were markedly improved with the introduction of anti-VEGF therapy. Adequate clinical trial design with selection of suitable endpoints constitutes a prerequisite towards clear assessment of additional potential therapeutic benefit by novel interventional approaches. Using machine learning algorithms, the present study outlines the possibility to predict retinal function, when (a) volumetric, multimodal retinal imaging data is obtained only or (b) additionally a short FCP exam is performed. For this AI-based analysis strategy, we have introduced the term “inferred sensitivity” that may serve as a functional surrogate endpoint in future clinical trials.

To date, BCVA constitutes the most common functional outcome parameter in clinical trials in ophthalmology in general and specifically in studies for neovascular AMD. However, BCVA is primarily representative of cone-function and of function at the fovea or of the preferred retinal locus in eyes with extrafoveal fixation (i.e. no spatial resolution)^4^. Moreover, BCVA assessment represents a psychophysical test that is rather time-consuming and requires good patient cooperation. FCP may partially compensate these shortcomings by allowing for differential testing of cone and rod function (dark-adapted FCP) and allowing for assessment of retinal loci outside the foveal center with moderate spatial resolution^11,12^. Disadvantages include the the duration of the examination, which limits the spatial resolution due to patient fatigue, and the need for specific equipment, i.e. a microperimetry device^14^. Hereby, surrogate endpoints represent a viable alternative to obtain quasi-functional results, especially with a high spatial resolution that could not be be achieved with psychophysical testing. This study demonstrates that it is possible to infer sensitivity based on routinely obtained structural imaging data. Using “inferred sensitivity” as a surrogate functional endpoint would provide five key advantages. “Inferred sensitivity” would (i) provide a much higher spatial resolution compared to current functional testing, (ii) be ubiquitously available and (iii) and could be obtained within a short time frame even in patient unfit for psychophysical testing. Moreover, (iv) “inferred sensitivity” could adequately represent potentially opposing treatment effect (e.g. edema reduction versus outer retinal atrophy), which would be inadequately represented by currently used SD-OCT surrogate endpoints such as central (full) retinal thickness. Finally, (v) “inferred sensitivity” could be compared across diseases to potentially facilitate objective cost-benefit analysis. All of these advantages would be relevant in interventional trials in neovascular AMD. Specifically, “inferred sensitivity” as an endpoint would allow for enrolment of patients with early extrafoveal or peripapillary CNV and/or concurrent macular atrophy in clinical trials^17–19^. These large subgroups of patients were previously systematically excluded from trials due to the limitations of BCVA as functional endpoint and of central [full] retinal thickness as a surrogate endpoint^17–19^.

Previous studies provided evidence for the close structure-function correlations between retinal sensitivity and multimodal imaging in AMD, albeit with only a limited number of narrowly selected predictors and/or application of linear models^7,8,20–24^. Building on this, by using a wide array of potentially predictive variables (26 imaging features) and non-linear models, it is demonstrated herein that the relationship between structure and function is indeed close. By electing a supervised machine learning approach using RF regression, we could evaluate the feature importance and graphically analyze the effect of these features. The fact that the ONL, which includes the cell bodies of the light-sensitive photoreceptor cells, was the most important feature for all three types of sensitivity predictions, underscores the biological plausibility of the models. However, importance of variables in the models may differ from biological relevance. Especially in the setting of correlated features, features exhibiting less measurement variability will be given higher importance. This may for example explain why the ONL thickness, which is significantly thicker than the IS and OS and therefore (relatively) less prone to grading errors, exhibited highest feature importance.

A similar observation has been previously reported in patients with intermediate AMD in absence of late-stage disease^25^. Interestingly, FAF intensity exhibited a high feature importance for mesopic and dark-adapted red test exceeding all of the SD-OCT features with the exception of ONL thickness. In contrast to SD-OCT features, the FAF intensity may be analyzed without any prior image segmentation, which is especially attractive for clinical evaluation. Various previous studies in AMD could demonstrate that FAF imaging allows not only for precise demarcation of geographic atrophy^26,27^, but may provide indirect information with regard to outer retinal thinning in the context of reticular pseudodrusen^28,29^, or loss of IS and OS in the context of persistently increased autofluorescence caused by prior subretinal fluid^30^.

The differential effect of retinal structure on mesopic versus dark-adapted cyan sensitivity further underscores the biological plausibility of our models. For example Fig. 3 shows that ONL thickening results in a more distinct reduction of dark-adapted cyan function as compared to mesopic function. Since all predictors were standardized in consideration of the location-specific normative values and since sensitivity losses rather than absolute sensitivity values were used as outcome variable, the inverted U-shaped for dark-adapted cyan testing may not be explained by the physiological rod photoreceptor distribution or ONL thickness topography. Accordingly, rod photoreceptor function appears to be more affected by macular edema as compared to cone photoreceptor function. Further, subretinal fluid or disintegrity of the RPE in terms of predictions leads to a more severe loss dark-adapted cyan sensitivity than mesopic or dark-adapted red sensitivity (Fig. 5). This would be in accordance with the observation that rod-photoreceptors are strictly dependent on the canonical visual cycle via the RPE, while cone-photoreceptors may obtain their chromophores via an additional cone-specific visual cycle involving Muller cells^31^.

Moreover, our study also included “patient-reliability indices” in the modeling process, demonstrating that consideration of these increased the prediction accuracy for scenario 1. In terms of interpretation, inclusion of these features appears to correct for patient-specific tendencies such as false positive responses.

Based on criteria established by the International Conference on Harmonization (ICH) Guidelines on Statistical Principles for Clinical Trials, “evidence for surrogacy depends upon (i) the biological plausibility of the relationship, (ii) the demonstration in epidemiologic studies of the prognostic value of the surrogate for the clinical outcome and (iii) evidence from clinical trials that treatment effects on the surrogate correspond to effects on the clinical outcome”^32^. While the biological plausibility is established in this study (as aforementioned), the other two aspects warrant further consideration. The second criterion is only partially applicable to “inferred sensitivity” given its quasi-functional character, in contrast to traditional surrogate endpoints that do not directly represent function (e.g. intra-ocular pressure in glaucoma). However, the third criterion is highly relevant for “inferred sensitivity” as a surrogate endpoint, since models are strictly limited by their applicability domain (i.e. predictor space where the model makes prediction with a given reliability). Clearly, the models developed here would be expected to perform sub-optimally in eyes with more rare forms of neovascular AMD including retinal angiomatous proliferation (RAP) due to the lack of corresponding training data in our cohort. In longitudinal clinical trials (third ICH criterion), it would be even more difficult to define the appropriate applicability domain as exemplified below.

### Limitations

Due to the exclusion of optic nerve diseases in our training data, the inner retinal features exhibit only a low feature importance in our models. The same consideration would apply for significant changes in media opacification (i.e. cataract). Therefore, “inferred sensitivity” based on our models would be unsuitable of reflecting certain potential side-effects such as optic neuropathy and glaucoma. To avoid such fallacies, at least a subset of patients in clinical trials, that evaluate change in “inferred sensitivity” as a surrogate endpoint, should undergo longitudinal FCP testing. Then, longitudinal accuracy of the models could be confirmed based on this subset prior to inferring sensitivity data for the remaining patients. Further, the discrepancy between the MAE of test and retest measurement differences versus the MAE for the prediction accuracy suggest that a larger training data set would have been beneficial (cf. Tables 1 and 2). Potentially, application of more complex AI approaches (e.g. convolutional neural network) to the raw imaging data could have further improved the prediction accuracies. However, the latter would have come at the cost of interpretability as well as an increased need for training data. Last, the study used the MAE as a conceptually simple and easily interpretable measure of model accuracy. Nevertheless, the root mean squared error, which penalizes particularly large errors that would be undesirable for inferred sensitivity, was also provided (Supplementary Table S2).

In summary, we have introduced the AI-based analysis strategy of “inferred sensitivity” to estimate differential effects of retinal structural abnormalities on cone- and rod-function in nAMD. This method constitutes a potential valuable tool to predict macular visual field losses at high-spatial resolution in future nAMD cohorts without the need for extensive psychophysical examinations. In the potential future application, individual subjects would undergo standard ophthalmological assessment and non-invasive retinal imaging in a relative rapid and straight-forward examination, while FCP testing only includes a limited number of test stimuli or can even completely be waived. The findings of this study suggest that “inferred sensitivity” opens the possibility for a refined investigation of treatment effects in nAMD superior to standard BCVA testing, particularly in order to differentiate functional outcomes of different treatment strategies. This technique may also be expanded in the future for high-resolution mapping of localized functional impairment in other macular and retinal conditions in order to investigate the functional impact of progressive structural abnormalities or to assess new therapeutical interventions. The notion of “inferred sensitivity” as a quasi-functional outcome measure might be further applicable to other retinal diseases including diabetic retinopathy, retinal vein occlusion as well as inherited retinal diseases.

## Methods

### Subjects

Subjects with neovascular AMD were recruited from injection clinics of the Department of Ophthalmology, University of Bonn. The inclusion and exclusion criteria have been published previously^33^. Inclusion criteria were age ≥50 years, a CNV lesion proven in OCT angiography (OCTA), fluorescein angiography (FA) and/or indocyanine green angiography (ICGA). Exclusion criteria for the study eye included refractive errors ≥5.00 diopters of spherical equivalent and >1.50 diopters of astigmatism assessed by autorefraction (ARK-560A; Nidek, Gamagori, Japan), a history of glaucoma or relevant anterior segment diseases with media opacities and no history of any intraocular surgery except cataract extractions <3 months ago. If both eyes met the inclusion criteria, the eye with better BCVA was included. Apart from taking the medical history, all subjects underwent routine ophthalmological examinations, including BCVA, slit-lamp and funduscopic examination. Control eyes were recruited from the hospital wards among patients with a healthy fellow eye and patient’s companions. The study protocol was in accordance with the relevant guidelines and regulations and approved by the Institutional Review Board of the University of Bonn (ethics approval ID: 191/16). Written informed consent conforming to the tenets of the Declaration of Helsinki was acquired from all participants.

### Imaging protocol

Based on previous publications, standardized retinal imaging was performed including combined confocal scanning laser ophthalmoscopy (cSLO) and spectral-domain optical coherence tomography (SD-OCT) imaging (30° × 25°, ART 25, 121 B-scans, Spectralis HRA-OCT 2, Heidelberg Engineering, Heidelberg, Germany)^33^. Further, 30° fundus autofluorescence (FAF) and multicolor imaging as well as 55° FAF imaging were performed on the same device. OCTA was performed using a swept-Source OCT (SS-OCT) device (3 × 3 mm, 6 × 6 mm, 9 × 9 mm OCTA scan, PLEX Elite 9000, Carl Zeiss Meditec AG, Jena, Germany). Color fundus photography (CFP) was performed (Visucam 500, Carl Zeiss Meditec AG). Both OCT-A and CFP were not included for prediction of inferred sensitivity.

### *Fundus-* controlled *perimetry*

FCP testing was carried out based on our previous experience with the S-MAIA (CenterVue, Padova, Italy) device in normal subjects and patients with intermediate and atrophic late stage AMD^11,13,14,25,33–35^. It was performed after dilating pupils using 2.5% phenylephrin and 0.5% tropicamide to facilitate fundus tracking. Patients with no prior perimetry experience underwent a short mesopic practice FCP test to accustom them to the procedure. Patients underwent duplicate (28 of 50 patients) or singular (22 of 50 patients) mesopic (achromatic stimuli, 400–800 nm) FCP, with subsequent 30 minutes of dark adaptation (light level <0.1 lux), followed by duplicate or singular dark-adapted cyan (505 nm) and dark-adapted red (627 nm) FCP using the S-MAIA device. Testing was performed with the pre-set 4–2 dB staircase strategy. The stimulus size was 0.43° (Goldmann III). The test grid consisted of 61 stimuli covering the central 18° of the retina. The test points were evenly distributed in five rings at 1°, 3°, 5°, and 9° around a central test-point. In terms of “patient-reliability indices”, false-positive responses were measured through presentation of suprathreshold stimuli to the optic nerve head (i.e. Heijl-Krakau method). Further, the rate of wrong pressure events was measured as the number of pressure events outside of the response window of the S-MAIA device^36^. Last, the 95% bivariate contour ellipse area (BCEA) encompassing 95% of the fixation points was recorded as measure of fixation stability^37^.

### Image analysis and grading

A proprietary approach for image analysis and non-linear registration was implemented as previously descriped^12^. Volumetric SD-OCT data were automatically segmented as implemented in the manufacturer’s software (Spectralis Viewing Module 6.3.2.0, Heidelberg Engineering, Heidelberg, Germany). Thereafter, the segmentation was reviewed and – if indicated - manually corrected by two consecutive readers. We defined all layers between the internal limiting membrane (ILM) and the outer plexiform layer (OPL) outer nuclear layer (ONL) boundary as inner retina^12,38^. Henle fiber layer (HFL) was counted towards the ONL in analogy to *Sadigh et al*.^12,39^. The inner photoreceptor segments (IS) ranged from band 1 (external limiting membrane [ELM]) to band 2 (ellipsoid zone [EZ]). The outer photoreceptor segments (OS) ranged from band 2 (EZ) to band 3 (putative interdigitation zone)^38^. The RPE-drusen complex (RPEDC) ranged from band 3 to Bruch’s membrane. As defined by *Chiu et al*., the RPEDC encompassed all drusen material, whether below the RPE (soft drusen and cuticular drusen) or above the RPE (reticular drusen, vitelliform debris)^40^. Subretinal fluid was included in the here used definition of the OS layer. The thickness from ILM to Bruch’s membrane was defined as full retinal thickness (Fig. 1).

Volumetric thickness maps for each layer were transferred as a tab-delimited file to ImageJ (U.S. National Institutes of Health, Bethesda, Maryland, USA) together with an outer retinal en-face image (mean intensity projection, 50 µm thick slab centered on IS/OS). To account for eye tilt and eye rotation between the FCP and SD-OCT examinations, the FCP data was registered to the outer retinal *en face* image using the moving least squares (non-linear) method (alpha 1.0, mesh resolution 64, affine transformation) as implemented in ImageJ (Fig. 1)^12^. FAF and IR images were aligned in the same manner. The mean thickness and reflectivity values (minimum-, mean- and maximum-intensity projection for each layer) of the volumetric SD-OCT data as well as the normalized FAF and IR intensity values topographically corresponding to the test-point locations and area (diameter of 0.43°) were semi-automatically extracted using ImageJ. In summary, 26 imaging features, as listed in supplementary table S1, were available for each test-point (FAF intensity, IR intensity as well as thickness, minimum- mean- and maximum-intensity projections for the SD-OCT layer [full retina, inner retina, ONL, IS, OS, RPEDC]).

### Statistical analysis

Statistical analyses were performed using the software environment R, version 3.5.1^41^. Visual acuity measurements were converted to the logarithm of the Minimum Angle of Resolution (logMAR). Data from normal eyes was used to obtain for each test-point (i.e. location-specific) normal sensitivity, layer-thickness and layer-reflectivity values. Hereby, linear regression analysis was used to obtain both, age-adjusted normative mean estimates and standard deviation estimates. The age was set to 76 years in consideration of the mean age of patients. Sensitivity measurements of patients were standardized by calculating the point-wise sensitivity loss as compared to the spatially corresponding normative value. If two sensitivity measurements were available for a patient, then the results of the test and retest were first averaged in a point-wise manner. Imaging features of patients were standardized by computing the z-scores (i.e. the number of normative standard deviations by which a given observation deviates from the normative mean estimate).

$${z}_{f,e,a}=\frac{{x}_{f,e,a}-{\bar{x}}_{{\rm{f}},{\rm{e}},{\rm{a}}}}{{S}_{{\rm{f}},{\rm{e}},{\rm{a}}}}$$

**Table:** 

**Symbol**	**Meaning**
*z*_*f,e,a*_	z-score for imaging feature (f) at the eccentricity (e) and angular position (a)
*x*_*f,e,a*_	Observation in a patient for imaging feature (f) at the eccentricity (e) and angular position (a)
${\bar{x}}_{{\rm{f}},{\rm{e}},{\rm{a}}}$	Age-adjusted normative mean value for a given imaging feature (f) at a given eccentricity (e) and angular position (a)
*S*_f,e,a_	Age-adjusted normative standard deviation for a given imaging feature (f) at a given eccentricity (e) and angular position (a)

For predictive modeling, we used Random Forest (RF) regression as implemented in the R packages *randomForest* and *caret*^42^. RF regression is a multivariable modeling technique that accounts for non-linear predictor-response relationships effects and possible interactions between predictors. In all models we used 1000 trees that were fitted to bootstrap samples of the training data. Two “outer” resampling techniques were used to assess the model accuracy. First, we used patient-wise leave-one-out cross-validation (LOO-CV) as scenario 1. Hereby, data of one patient (61 observations) served as test set, while the data of the remaining patients (49 patients with 61 observations each) served as training set. This procedure was repeated until the data of every patient served as a test set exactly once (i.e. 50 folds, cf. Fig. 2). Further, we used test-point-wise CV by additionally adding half of the perimetry data (30 or 31 observations) of a given patient to the training set and using only the remaining half of the perimetry data of that given patient as test set as in scenario 2. Again, this procedure was repeated until each half-dataset of every patient served as a test set exactly once (i.e. total of 100 folds, Fig. 2). The parameter *mtry* (denoting the number of candidate variables at each split of the procedure) was optimized using the *tuneGrid* argument in *caret* probing the values of 4, 6, 8, 10 (scenario 1) or 40, 50, 60, 70 (scenario 2) using “inner” resampling (5-fold CV) on the training data. For both scenarios (S1, S2), three sets of predictor variables were evaluated: (A) imaging data only (26 candidate variables), (B) imaging data and “patient reliability indices” (30 candidate variables) and (C) imaging data, “patient reliability indices” and fixation stability (31 candidate variables). Moreover, for all variants of scenario 2, the eye ID was added by one-hot encoding as predictor. The mean absolute difference (MAE) between predictions and measured observations was used as measure of prediction error (additionally, root mean *squared* error [RMSE] estimates are presented in Table S2). Hereby, the MAE and 95% confidence interval for the MAE estimates were calculated considering the hierarchical structure of the data (test-point nested in eye). The MAE values for the retest-variability for the subset of patients with two tests were provided as benchmark. Further, the MAE values for null models, which constitute covariate-free models with a single numeric outcome, were provided for both scenarios. For Scenario 1 the null model was fitted using patient-wise LOO-CV and thus produces as output the mean sensitivity loss of the training data of each fold. For Scenario 2, the null model produces the patient-specific mean obtained by half of the perimetry data to predict the sensitivity losses for the other half of the data (cross-validated with 100 folds [2 per patient]). The prediction accuracies were compared with mixed models using the R package *lme4*^43^. Hereby, the prediction accuracy (absolute errors) was considered as outcome variable and test-points nested in patients as random effect. First, a “global” likelihood ratio test was used to assess, whether the model type (i.e. S1A, S1B […] S2C) had an influence as fixed effect on the prediction accuracy. Then, post-hoc pair-wise comparisons (Tukey contrasts) were used to determine which MAE values differed significantly from each other significantly. The feature importance was evaluated based on permutation of out-of-bag (OOB) data and measured as percentage of increase in mean *squared* error (% Inc MSE). For visual inspection of the models, we used the R package *forestFloor*^44^. This package allows for plotting of the out-of-bag cross validated feature contributions (y-axis) in dependence of each predictor variable (x-axis). Further, a measure of goodness-of-visualization (R^2^) is provided, to describe the variance of the feature contribution for a given predictor explained by the plot.

## Supplementary information

Supplementary table S1 + S2

## Data Availability

The datasets generated during and/or analyzed during the current study are available from the corresponding author on reasonable request.
