# Supplementary material for: Artificial intelligence for morphology-based function prediction in neovascular age-related macular degeneration
Source: Sci Rep. 2019 Jul 31;9:11132. doi: 10.1038/s41598-019-47565-y (PMC6668439; doi:10.1038/s41598-019-47565-y)
Supplement: Supplementary file 1 — Supplementary table S1 + S2 [file 41598_2019_47565_MOESM1_ESM.docx]

**Artificial intelligence for morphology-based function prediction in neovascular age-related macular degeneration**

*Leon von der Emde^#1^, Maximilian Pfau^#1,2^, Chantal Dysli^1,3^, Sarah Thiele^1,2^, Philipp T. Möller^1,2^, Moritz Lindner^4^, Matthias Schmid^5^, Monika Fleckenstein^1,2^, Frank G. Holz^1,2^, Steffen Schmitz-Valckenberg*^1,2^*

*# These authors contributed equally to this work.*

** Corresponding author*

1. Department of Ophthalmology, University of Bonn, Ernst-Abbe-Str. 2, Bonn, Germany
2. GRADE Reading Center, Ernst-Abbe-Str. 2, Bonn, Germany
3. Department of Ophthalmology and Department of Clinical Research, Inselspital, Bern University Hospital and University of Bern, Bern, Switzerland
4. The Nuffield Laboratory of Ophthalmology, Sleep and Circadian Neuroscience Institute, Nuffield Department of Clinical Neurosciences, University of Oxford, Oxford, United Kingdom
5. Institute for Medical Biometry, Informatics and Epidemiology, University of Bonn, Germany

**Correspondence:**

Professor Dr. Schmitz-Valckenberg

Department of Ophthalmology

University of Bonn

Ernst-Abbe-Str. 2

53127 Bonn

Germany

Tel.: +49 228 287 16826
Fax: +49 228 287 11470

E-Mail: [steffen.schmitz-valckenberg@ukbonn.de](mailto:steffen.schmitz-valckenberg@ukbonn.de)

**Supplementary Table S1. Definition of predictive features**

Abbreviations: Full retinal thickness (FRET); inner retinal thickness (IRET); outer plexiform layer (OPL); outer nuclear layer (ONL); photoreceptor inner segments (IS); photoreceptor outer segments (OS); retinal pigment epithelium-drusen complex (RPEDC); Bruch's membrane (BM)

| **Feature type** | **Feature** | **Feature description** |
| --- | --- | --- |
| **Imaging features** | FRET thickness | Axial thickness between ILM and BM |
|  | FRET max. intensity | Maximum reflectivity between ILM and BM |
|  | FRET mean intensity | Mean reflectivity between ILM and BM |
|  | FRET min. intensity | Minimum reflectivity between ILM and BM |
|  | IRET thickness | Axial thickness between ILM and OPL/ONL boundary |
|  | IRET max. intensity | Maximum reflectivity between ILM and OPL/ONL boundary |
|  | IRET mean intensity | Mean reflectivity between ILM and OPL/ONL boundary |
|  | IRET min. intensity | Minimum reflectivity between ILM and OPL/ONL boundary |
|  | ONL thickness | Axial thickness between OPL/ONL boundary and ELM |
|  | ONL max. intensity | Maximum reflectivity between OPL/ONL boundary and ELM |
|  | ONL mean intensity | Mean reflectivity between OPL/ONL boundary and ELM |
|  | ONL min. intensity | Minimum reflectivity between OPL/ONL boundary and ELM |
|  | IS thickness | Axial thickness between ELM and EZ |
|  | IS max. intensity | Maximum reflectivity between ELM and EZ |
|  | IS mean intensity | Mean reflectivity between ELM and EZ |
|  | IS min. intensity | Minimum reflectivity between ELM and EZ |
|  | OS thickness | Axial thickness between EZ and RPEDC inner boundary |
|  | OS max. intensity | Maximum reflectivity between EZ and RPEDC inner boundary |
|  | OS mean intensity | Mean reflectivity between EZ and RPEDC inner boundary |
|  | OS min. intensity | Minimum reflectivity between EZ and RPEDC inner boundary |
|  | RPEDC thickness | Axial thickness between RPEDC inner boundary and BM |
|  | RPEDC max. intensity | Maximum reflectivity between RPEDC inner boundary and BM |
|  | RPEDC mean intensity | Mean reflectivity between EZ and RPEDC inner boundary and BM |
|  | RPEDC min. intensity | Minimum reflectivity between RPEDC inner boundary and BM |
| **Patient reliability indices** | Exam duration | Duration of the fundus-controlled perimetry examination |
|  | False Positive rate | Rate of false-positive responses (FP) to suprathreshold stimuli presented to the optic nerve head (Heijl-Krakau method) |
|  | Wrong pressure events | Number of pressure events outside of the response window of the fundus-controlled perimetry device (S-MAIA, CenterVue, Italy) |
| **Fixation stability** | Log10(95%BCEA) | Common logarithm of the 95 % bivariate contour ellipse area (BCEA) |

**Supplementary Table S2. Root mean square errors**

The root mean *squared* error (RMSE) estimates in dB and the 95% confidence interval for the RSME estimates are provided as additional measure of prediction error (cf. Table 2).

|  | **Scenario 1 (LOO-CV on patient-level)** | | | | **Scenario 2 (LOO-CV on test-point level)** | | | |
| --- | --- | --- | --- | --- | --- | --- | --- | --- |
|  |  | **Random forest regression** | | |  | **Random forest regression** | | |
| **Type of testing** | **Null model** | **Imaging data** | **Imaging data and reliability indices** | **Imaging data and reliability indices and fixation stability** | **Null model** | **Imaging data** | **Imaging data and reliability indices** | **Imaging data and reliability indices and fixation stability** |
| **Mesopic (RMSE in dB)** | 6.49 [5.66, 7.32] | 5.27 [4.67, 5.87] | 5.1 [4.45, 5.75] | 4.9 [4.27, 5.53] | 4.67 [4.04, 5.3] | 4.09 [3.71, 4.47] | 3.7 [3.32, 4.07] | 3.66 [3.29, 4.03] |
| **Dark-adapted cyan (RMSE in dB)** | 7.62 [7.2, 8.04] | 6.18 [5.68, 6.69] | 5.87 [5.49, 6.25] | 5.9 [5.53, 6.28] | 5.89 [5.54, 6.24] | 4.98 [4.69, 5.28] | 4.64 [4.34, 4.95] | 4.62 [4.31, 4.93] |
| **Dark-adapted red (RMSE in dB)** | 6.37 [5.68, 7.05] | 4.95 [4.5, 5.39] | 4.96 [4.5, 5.42] | 4.84 [4.42, 5.27] | 4.69 [4.25, 5.12] | 3.97 [3.66, 4.28] | 3.73 [3.45, 4.01] | 3.62 [3.35, 3.9] |
